# The Journal Banquet—A Surprise

**Published:** 1901-05

**Authors:** 


### THE JOURNAL BANQUET—A SURPRISE.

THE Editors of the JOURNAL OF COMPARATIVE MEDICINE AND VETERINARY ARCHIVES had the pleasure of entertaining the members and visitors attending the annual meeting of the Pennsylvania Veterinary Medical Association at a dinner in the dining hall of the University of Pennsylvania on the evening of the last day's session, March 6th.

Dinner was served at 9 P.M.

MENU.

Consomme Julienne. Fried Smelts, Tartar Sauce. Saratoga Chips.

Sweetbread Croquettes.

Roast Turkey. Mashed Potatoes. Green Peas. Stewed Tomatoes.

Lettuce and Tomato Salad.

Bisque Ice Cream. Mixed Cake.

Coffee. Cigars.

The following guests were present: Drs. U. S. G. Bieber, of Kutztown; J. N. Becker, Palmyra; W. G. Benner, Doylestown; F. Bridge, Philadelphia; John L. Bradley, Mercersburg; B. F. Bachman, Strasburg; A. O. Cawley, Milton; J. M. Carter, Philadelphia; E. L. Cornman, Philadelphia; John M. Courtright, Clark's Green; M. J. Collins, Myerstown; Chas. H. Detwiler, Iron Bridge; John C. Foelker, Allentown; W. H. Fry, Pine Grove Mills; John T. Ferley, Jr., Philadelphia; J. O. George, Camden, N. J.; Alexander Glass, Philadelphia; Chas. T. Goentner, Bryn Mawr; Robert Gladfelter, Philadelphia; C. R. Good, Lockhaven; S. J. J. Harger, Philadelphia; J. D. Houldsworth, Manayunk, Philadelphia; W. Horace Hoskins, Philadelphia; Rush S. Huidekoper, Washington, D. C.; Jacob Helmer, Scranton; J. B. Irons, Erie; Geo. B. Jobson, Oil City; H. S. Jackson, Sewickley; A. G. Kern, Philadelphia; Jos. Johnson, Kirkwood; Z. S. Keil, Perkasie; Wm. Herbert Lowe, Paterson, N. J.; G. Magee, Uniontown; J. C. McNeil, Pittsburg; C. J. Marshall, Philadelphia; L. E. Meade, Tunkhannock; A. R. May, Boiling Springs; M. Moriarty, Gettysburg; Harry T. McNeal, Shickshinny; J. W. Mather, Rohrersburg; Chas. E. Magill, Philadelphia; E. W. Newcomer, Mt. Joy; Otto G. Noack,

Reading; J. H. Oyler, Harrisburg; J. R. Phillips, Library; Leonard Pearson, Philadelphia; E. C. Porter, New Castle; E. W. Powell, Oxford; A. R. Pottiger, Selinsgrove; Warren L. Rhoads, Lansdowne; E. M. Ranck, Philadelphia; N. Rectenwald, Pittsburg; A. F. Schreiber, Philadelphia; J. W. Sallade, Pottsville; W. J. Storm, Philadelphia; A. T. Sellers, Camden, N. J.; S. J. Swift, Franklin; E. E. Tower, Montrose; Henry W. Turner, Lahaska; W. J. Waugh, Pittsburg; Jas. B. Weir, Smicksburg; A. W. Weir, Greenville; Messrs. Harry E. Bender, Harry Chain Bassler, Chas. L. Colton, J. W. Hayman, O. M. Norton, Reuben Rivers, C. Scott Shore, Harry W. Watson, Frank J. Woodward, students in attendance at the Veterinary Department of the University of Pennsylvania, and H. L. Anglock.

Letters and telegrams of regret were received from Prof. McEachram, of Montreal; Profs. A. Smith, D. K. Smith, and Sweetapple, of Toronto; Profs. Lyman, Osgood, and Sheldon, of Boston; Profs. Roscoe R. Bell and Robertson, of New York; Profs. Baker and Hughes, of Chicago; Profs. McKillip and Merillat, of Chicago; Dr. Dougherty, of Baltimore; Dr. D. E. Salmon, of Washington; Dr. J. L. Winchester, of Lawrence, Mass.; Dr. L. H. Howard, of Boston, and many others.

At the conclusion of the dinner, as the hosts were about to call for toasts from their distinguished guests, the floor was taken from them by Dr. Leonard Pearson, who placed upon the table in front of Dr. W. Horace Hoskins, the managing editor of the JOURNAL, a superb oak wood box, containing a magnificent dinner set of silver, knives, forks, and all sizes of spoons, in double dozens. Dr. Pearson then addressed the bewildered editor as follows:

### *Remarks of Dr. Pearson.*

"There is one of whom I wish to speak—I would say a few words of one of our number who was born not far from here, whose whole life has been passed in our midst and whose career is an open book—Dr. Hoskins.

"Dr. Hoskins has been spoken of in my presence as a self-made man. But this designation as applied to him is far from the truth. Our friend came into the work with the best endowment and under the most favorable auspices that could have been contrived. He was heavily endowed with vigor of mind and body, with energy, with ambition, with high ideas, and with brains. He was nurtured under conditions that favored the good traits of his character. If further proof of this is wanted, one need but see the other plants that grew from the same stock and in the same garden.

"How immeasurably better is this endowment and those home conditions than are stacks of gold and an early life of idleness and luxury! How much more is the barefoot country boy of rugged body and thrifty race to be envied than the pampered son of the millionaire! and how much more the world owes to these children of nature and of God than to the sluggish offspring of empty riches!

"Fortunately for himself, for his family and for us, Dr. Hoskins had within him that which enabled him to keep within the straight and narrow path upon which he was so well started, to develop, to expand, to advance and finally to arrive.' Dr. Hoskins has arrived. He has arrived at a place in our esteem and affection that is his alone. Let me recite briefly a few of the events that have served to give our host the unique position he occupies. I shall refer only to his career since he began to study the veterinary science. As an undergraduate he was ever active in pressing for union and fellowship among his classmates. He constantly stood for improvement of the institution with which he was connected. His spirit even then was constructive. After graduation he became active in the Alumni Association of his Alma Mater and helped to place the American Veterinary College on a plane above most of the other proprietary schools, where it stood for professional advancement even to its own serious financial injury. He was one of the organizers, and has never missed a single meeting of this State Society. He has served as Secretary and President and Trustee and Committeeman, and, indeed, in every position of trust and responsibility that the Association has had; and in each place he has done faithful work, has established a new record of achievement and has made a precedent harder for his less vigorous successor to follow. As a founder and unremitting supporter of the Keystone Veterinary Association he has been equally constant.

"In 1884 the United States Veterinary Medical Association was languid and latent. Dr. Hoskins then joined that Society. Within a short time, under his invigorating example and energetic leadership, it awoke, it took on new life and is now a truly national one, and, indeed, an international organization. As Secretary and President of that organization our friend has led it to its greatest triumphs. When the history of veterinary education in America is written a long and interesting chapter can be devoted to the efforts—and the successful efforts—of our colleague to place the schools on a three-year basis; how he made this a condition for membership in the

United States Veterinary Medical Association and for practice in the States; how he antagonized and fought his fellow alumni and his former teachers; how he defeated them and, finally, how they admitted the wisdom of his views and blessed him. There is scarce time even to mention the value of his leadership and help in obtaining the passage of the various laws governing the rights of veterinarians to practice in Pennsylvania, for the control of the diseases of animals and of his efforts to secure a purer supply of meat and milk. Think of any good thing that has been done for public veterinary medicine during the past seventeen years, follow it back to its origin and you will find Dr. Hoskins working like a beaver to give it a firm foundation, wholesomeness and impetus.

"But it is not in veterinary affairs alone that our friend has rendered valued service. He has always been active in the cause of good government, and has been a most active worker for civil service and ballot reform. He has led his party in one of their contests in this city and has become prominent as an advocate of some issues of national politics. As a director of several large business corporations he has shown the manysidedness of his character, and has proved that his development has not been in one direction at the expense of ability in others—but that his principles are foundation principles and will carry their posessor successfully in any direction he wills.

"As a veterinary journalist he now leads all America; as a Mason, he has reached the height of many men's ambition; as a successful practitioner of veterinary medicine, he is at the head of the profession in this city.

"But it is in Dr. Hoskins' personal relations with his colleagues and in his home life that his truest metal is shown—where he stands not as a doer of deeds but as a man—and that he becomes most attractive.

"But into these admirable relations it is not our place to enter. Now we are thankful to have such a man among us—we are grateful to him for his help to us, and we feel that we wish him to know that it is appreciated. Such unselfish devotion is beyond the reach of reward except "as seed sown by the wayside may fall upon good ground and spring up to bear fruit." And so to-night the veterinarians of Pennsylvania bring to you, sir, this offering as a concrete expression of the kind feelings of your many friends, and we beg you to accept it with our best wishes for your continued prosperity and happiness."

For once in his life Dr. Hoskins' nerve gave way, and a grasp of the hand to his many friends who had contributed to this surprise had to take the place of words.

